# Gold Dust and Iron Filings as an Antidote for Corrosive Sublimate

**Published:** 1863-06

**Authors:** Christopher Johnston

**Affiliations:** Baltimore, Md.


### Selections.

---

On Gold Dust and Iron Filings, as an Antidote for Corrosive Sublimate.—*By Christopher Johnston, M. D., Baltimore, Md.*—In the year 1841, a rejected lover, at that time a visitor in Baltimore, committed suicide by taking a large dose of the corrosive chloride of mercury. The case fell into the hands of Dr. Thomas H. Buckler, who employed, unavailingly, all the known antidotes for this destructive agent, and had the misfortune to see his patient die in great agony. The failure of art to relieve made a strong impression upon Dr. Buckler, and he forthwith instituted experiments with the view of ascertaining by observation the efficacy and value of the various articles used or proposed to counteract the poisonous effects of the mercurial salts.

In the course of these experiments upon pigs and dogs, it occurred to him to magnify the *galvanic test* into an antidote —for, said he, if the corrosive chloride in solution, being placed on a bright gold surface, and touched with an iron point which is also brought in contact with the gold, undergoes decomposition, there is no reason why gold and iron in the form of powder, as exposing great surface, should not also separate chlorine and mercury in combination in the living stomach. Besides, the elements are instantly appropriated by the antidotal agents, "the mercury attaching itself to the negative electrode, namely, the gold, while the chlorine unites with the iron of the positive electrode to form chloride of iron; and thus, for a highly dynamic substance, we substitute a comparatively inert amalgam of gold and a harmless chloride of iron."

Accordingly into the stomach of pigs and dogs he introduced poisonous doses of the corrosive chloride of mercury, taking care to control the œsophagus; and then, after various intervals, he passed an estimated quantity of gold dust and iron filings into the stomach of a portion of the animals. All the poisoned animals which had not received the antidote became the victims of the experiment; and of the others, those only died which had presented very severe symptoms before the administration of the remedy.

These results were published by Dr. Buckler in the Baltimore *Medical and Surgical Journal* in 1843, with a recommendation, by the author, of the *new antidote* which he proposed. But the suggestion seemed to have received no attention whatever, until, in the last year, a case of corrosive sublimate poisoning occurred to us, in the which we essayed for the first time gold dust and iron filings upon the human subject.

We had had the good fortune to witness a repetition of Dr. Buckler's experiments, and were familiar with his views in respect of the agency at work; so that we only awaited a fitting opportunity for testing the value of our friend's proposition.

On the 14th of May last, a gentleman of this city, being disturbed in mind, procured two drachms (ʒ ij.) of corrosive sublimate from an apothecary, assigning as a reason his intention of destroying rats; mixed two-thirds of the salt with whiskey and water and swallowed the whole at a draught, leaving no dregs in the tumbler. In about ten minutes his wife, hearing efforts at vomiting in her chamber, proceeded thither, and found her husband sitting up in violent and agonizing emesis. A word and the fatal paper satisfied her as to the danger of the sufferer; whereupon she despatched a messenger for medical aid, but administered the while milk and white of egg, having in a previous marriage been the observant wife of a physician.

In five minutes—it so happened—we were at the bedside, and the galvanic antidote being present in our mind, we sent in haste for two drachms of iron by hydrogen, and a book of gold-leaf. While waiting for the arrival of the articles we encouraged perseverance in the use of albumen and milk, but the patient continued to vomit freely, violently, and unintermittingly. Everything swallowed was rejected as soon as it approached the stomach, and then, after sturdy efforts, small quantities of greenish mucus, streaked with blood, were discharged. The face was much congested, the body cold, and the whole surface bedewed with sweat.

In less than ten minutes (in all, somewhat short of twenty-five minutes from the ingestion of the sublimate) we had prepared a bolus by dusting the surface of a leaf of gold with the iron, over this another leaf, then iron, and so on, alternating the two metals until about one-half of the gold leaf had been expended, and the mass was rolled into a ball.

Before swallowing this, however, we administered warm water to effect the removal of mucus, albumen and milk, should any of these matters be in the stomach, as they must defend the sublimate from the action of the antidote by preventing contact. Instant emesis followed.

The bolus was readily taken into the stomach, aided by a little water, for the poisoned man regretted his act, and wished to escape from death. For five minutes there was a calm, during which we made another bolus with the remainder of the gold leaf and iron.

Vomiting now recurred, but with less violence, and the matters ejected were tinged with yellow or light brown, and contained particles of gold leaf.

Presently another moment of quiet occurred, which was the signal for the administration of the second bolus. Vomiting now ceased entirely, although at intervals for an hour a slight tendency towards emesis was observable—but the frantic, tremulous anxiety had given place to confidence and composure—the man assumed a hopeful appearance, and we were of opinion that the first—the greatest danger—was past.

On the next day, moderately severe ptyalism manifested itself; but the convalescence was rapid, and the cure was complete in about eight days.

The after-treatment, when vomiting was arrested, was very simple, consisting of hydrocyanic acid in mucilage with small doses of morphia; mucilaginous drinks, as of gum arabic, or quince seed, were directed; and beef-tea and arrowroot were the essential articles of diet for some days. Rochelle salt dissolved in soda water was given on the first days, and counter-irritation over the epigastrium practiced from the beginning, and continued until all signs of gastric disturbance had disappeared. And, to conclude the statement, the mouth symptoms were met by gargles of tinct. perchloride of iron largely diluted with water, alum in infusion of sage, and lastly, flaxseed tea acidulated with lemon.

We would not insist upon the superior efficacy of gold leaf and of iron by hydrogen over gold dust and fresh iron filings —although in the case reported the virtue of the former was apparent to and acknowledged by all the witnesses—for we have still a preference for the latter, on account of the freshness of the metallic surfaces. But in the hurry of the moment, the first mentioned may be easily obtained in a state of preparation excepting on Sundays, and then even a dentist would furnish gold foil, No. 4 or lighter, which would supply the placc of gold leaf. If the conveniences were at hand, or, if we were near a pharmaceutist's, we would prefer to grind gold-foil with fresh iron filings in a mortar, and exhibit the coarse powder so produced with a little water; for the heavier particles would more quickly find their way to the mucous coating of the depending portion, while enough of the finer particles would distribute themselves throughout the stomach to accomplish the destruction of the corrosive chloride not in contact with the mucous membrane.

With regard to the quantity and proportions of gold and iron to be administered together as an antidote for the corrosive mercurial salt, it is desirable to know approximately the amount of the poison taken; but this is not indispensable for an overdose of the remedy would occasion no "unpleasant" consequences. Lest an insufficient quantity of the metals be exhibited, a reference to the table of equivalents is necessary. As for mercury and gold there is much discrepancy among authors, but if we adopt for Hg 100.1 (Erdmann) for Au 196.44 (Berzelius), for Fe 28.04 (Erdmann) and for Cl 35.5 (Berzelius), we have for corrosive sublimate the formula Hg Cl (100.1+32.5)=135.6, and 100 grains would contain Hg 73.82, and Cl 26.18. Now, since the corrosive sublimate in the presence of gold and iron is reduced, and there are formed an amalgam of gold and sesquichloride of iron, we must know how much iron is needed to make that compound with Cl 26.18. The formula for the sesquichloride is $Fe_2Cl_3$ (56.08+106.5)=162.58; therefore in 39.96 grains we have Cl 26.18 combining with Fe 13.78, the quantity sought.

Again, to estimate the gold, assuming the amalgam formed to be in equivalent proportions, we find AuHg (196.44+100.1)=296.54; and in 218 grains we discover 144.86 grains of the precious metal, and 73.82 grains of mercury. Or, if we employ the equivalents adopted by Cahours, thus, Au 98.18 (not far from Graham, Au 99.6) and Hg 103, we have 140.92 grains of amalgam yielding 67.10 grains of gold. In short, we need 144.86 grains of gold (or else 67.10 grains) to appropriate the mercury liberated by the action of the reagents. This discrepancy is not likely to lead to ill results, for, if we use the larger number, we might be quite at ease about the amalgam produced, and it is not certain that the lesser quantity would contribute to form an amalgam more nocuous than the other.

To sum up: For 100 grains of the corrosive sublimate of mercury taken, there would be required of iron 13.78 grains, or rather less than the one-seventh part by weight, and of gold dust 144.86 grains, or about one-and-a-half times the weight, to effect the decomposition of the poison, and the complete appropriation of its elements. If we follow Cahours we need only employ 67.10 grains of gold.

In conclusion we would remark, that very large doses of the antidote must be rarely needed, since vomiting, which marks the earliest effects of the corrosive chloride of mercury necessarily rids the stomach of some portion of the destructive agent.—*Am. Journal of the Medical Science.*

